# Editorial: Transport in Plant Microbe Interactions

**DOI:** 10.3389/fpls.2016.00809

**Published:** 2016-06-08

**Authors:** Pierre-Emmanuel Courty, Daniel Wipf

**Affiliations:** ^1^Department of Biology, University of FribourgFribourg, Switzerland; ^2^UMR 1347 Agroécologie, BP 86510, Centre National de la Recherche Scientifique, Institut National de la Recherche Agronomique, University of Bourgogne Franche-ComtéDijon, France

**Keywords:** transportome, transport, plant–microbe interactions, membrane transport proteins, membrane

Plant–microbe interactions are omnipresent in terrestrial ecosystems and central to understand processes of individual growth, community assembly, and biogeochemical cycling. Plants and microbes interact above and below ground, and such interactions could theoretically include all combinations of positive (i.e., mycorrhizal and legume-rhizobia), negative (i.e., pathogenic interactions), or neutral effects. Many plant pathogenic and symbiotic microbes produce specialized structures that invade plant cells, but remain enveloped by plant-derived membranes. These intimate contacts between plant and microbial structures drive either bidirectional flows of nutrients as symbiotic (mycorrhizal or legume-rhizobia) or unidirectional flows as in pathogenic interactions. Whatever the biotrophic context (symbiotic vs. pathogenic), nutrients must pass several membrane barriers and the apoplastic interface before their assimilation by plant or microbial cells. Plant and microbial cells must be “re-programmed,” which includes differentiation and polarization of membrane transport functions to take up, to transfer or to exchange nutrients between partners of the biotrophic interaction. However, the mechanisms underlying the functioning and the dynamics of the transportome (the range of genes of an organism that encode proteins contributing to transport molecules across cellular membranes: membrane transporters, ions exchangers, and ion channels) at the biotrophic interface are still poorly understood. The transportome is a key player in nutrient uptake and exchange mechanisms and its regulation pattern is essential in determining the outcome of plant fungal interactions and in adapting to environmental changes.

Availability, uptake, and exchange of nutrients in biotrophic interactions will drive plant growth and modulate biomass allocation, that are central to plant yield, a major outcome, in the context of high biomass production. In a long term approach, unraveling those biotrophic transportomes and their underlying mechanism will be extremely useful in (i) the prediction of plant–microbes ecological niches, (ii) plant diagnosis (i.e., health, nutritional status), (iii) our understanding of microbial ecology and evolution of function, (iv) the development and implementation of environmentally and sustainably agro-ecosystems for crop production, (v) the identification of natural routes to the cycling and sequestration of carbon in terrestrial environments, and (vi) the ecosystem response to climate change (i.e., Schroeder et al., 2013; Gerlach et al., 2015; Larsen et al., 2015; Lemanceau et al., 2015).

Comparative genomics revealed that plants and microbes have a variable repertoire of transporters (Ward et al., 2009; Kohler et al., 2015). In prokaryotes, organisms with larger genomes have been shown to have proportionally more transporters (Paulsen et al., 2000; Markowitz et al., 2012). The question of number of microbial transporters functioning at the biotrophic interface is of central interest. This number could be related either to (i) a strong host dependency, (ii) a reduced host dependency if the genome complexity increased, or (iii) a broad plant range with which they interact. The nutritional/trophic transportome puzzle at the biotrophic interface is still far from complete and major pieces such as (i) the system of cellular efflux are still missing, (ii) the functional regulation within microbial species is a black box, (iii) the regulation of nutrient exchanges between organisms is still poorly understood, (iv) the knowledge on the alteration/reorganization of the traffic of vesicular membranes in both partners is on infancy (i.e., Leborgne-Castel and Bouhidel), and (v) also the metabolic patterns of how plants and microbes interact at the biotrophic interface is unknown, suggesting that the key transporter genes need to be elucidated from model organism. Regarding the availability of plant and microbial genomes, only several transporters involved in biotrophic interactions were characterized: mycorrhizal symbiosis (phosphorus nutrition: Becquer et al. general overview: Casieri et al., 2013; nitrogen nutrition: Courty et al., 2015; phosphorus nutrition: Garcia and Zimmermann), root nodule symbiosis (Clarke et al.), actinorhizal symbiosis (Imanishi et al.), and pathogenic interaction (amino-acid nutrition: Struck). Moreover, only few recent studies are about such characterization: metal transporters (Tamayo et al.), an ammonium transporter (Calabrese et al.), phosphate transporters (Walder et al., 2016), and a dipeptide transporter (Belmondo et al.) in the mutualistic fungus *Rhizophagus irregularis* forming arbuscular mycorrhizas, an aquaporin (Xu et al., 2015) in the mutualistic fungus *Laccaria bicolor* forming ectomycorrhizas, a monosaccharide transporter (Schuler et al., 2015) in plant pathogenic fungi *Ustilago maydis*, phosphate transporters (Walder et al., 2015), monosaccharide transporters (Doidy et al., 2012), and sulfate transporters (Casieri et al., 2012) in mycorrhizal plants and an hexose transporter in plants infected by pathogens (Moore et al., 2015). Effects of nutrient deficiency on the transcriptome of both partners at the biotrophic interface are poorly characterized (Bonneau et al., 2013; Wipf et al.). Increasing applications and improvements of methodologies could complete classical transporter characterization and give a better detection and resolution of the functioning of biotrophic interfaces: transcripts and proteins by laser capture microdissection technology (Koegel et al., 2013), nutrients by NanoSIMS (Kaiser et al., 2015), and metabolites by liquid/gas chromatography–mass spectrometry (Gaude et al., 2015; Rivero et al.). These recent technological achievements in model plants associated to microbial consortia will facilitate comprehensive identification of the key nutrient transporters involved in biotrophic (mutualistic and pathogenic) interactions. The functioning of some of these transporters (i.e., phosphate transporter) could be evolutionary linked in plant–mutualistic (Delaux et al., 2013) and plant–pathogen (Wirthmueller et al., 2013) interactions.

Beside the biotrophic interactions, we should also consider interactions between microbes and their environment (rhizosphere, phyllosphere) that could influence microbial nutritional/trophic transportome. Soils, minerals, and leaves represent specific microbial habitats influencing and controlling the establishment of microbial communities, but also the expression of transport-related genes (Johnson, 2010; Correa et al., 2015; Uroz et al., 2015).

## Author contributions

PC and DW have co-edited the topic and co-prepared the editorial.

### Conflict of interest statement

The authors declare that the research was conducted in the absence of any commercial or financial relationships that could be construed as a potential conflict of interest. The handling Editor declared a shared affiliation, though no other collaboration, with one of the authors P-EC and states that the process nevertheless met the standards of a fair and objective review.
